# Primary caregiver knowledge of paediatric physical activity recommendations in the United Kingdom and its association with caregiver behaviour: an observational study

**DOI:** 10.1186/1471-2458-14-795

**Published:** 2014-08-04

**Authors:** Alexia Sawyer, Lee Smith, Stephanie Schrempft, Cornelia HM van Jaarsveld, Jane Wardle, Abigail Fisher

**Affiliations:** Department of Epidemiology and Public Health, Health Behaviour Research Centre, University College London, Gower Street, London, WC1E 6BT UK; Department of Primary Care and Public Health Sciences, King’s College London, Capital House, 42 Weston Street, London, SE1 3QD UK

**Keywords:** Physical activity, Physical activity guidelines, Children, Primary caregivers

## Abstract

**Background:**

Most children in affluent developed countries do not meet basic physical activity recommendations. This study assessed primary caregiver knowledge of the UK recommendations on physical activity for children and examined the relationship between knowledge and components of parental support and modelling of physical activity.

**Methods:**

Data were from a large, community-based twin birth cohort. Primary caregivers were invited to take part in a telephone interview on the home food and activity environment that included a question on knowledge of the minimum amount of physical activity recommended in UK child guidelines. Socio-demographic variables (maternal age, BMI, education, ethnicity and presence of co-habiting partner) were available from previously completed questionnaires. Parental support and modelling of physical activity variables were assessed during the telephone interview. Binary logistic regression analyses examined the relationship between knowledge and socio-demographic variables and components of parental support and modelling of physical activity.

**Results:**

1,113 families took part in the interview. Only 21% of participants knew the recommended amount of physical activity for children. Higher maternal education was associated with knowledge of the recommendation (Odds Ratio (OR) 2.82; 95% Confidence Interval (CI) 1.66, 4.79, p < 0.001). Knowledge of the recommendation was associated with communicating positive messages about physical activity to child (OR 1.52; 95% CI 1.12, 2.06, p = 0.008), watching the child participating in physical activity (OR 1.69; 95% CI 1.11, 2.55,p = 0.013) and showing the child they enjoyed physical activity themselves (OR 1.51; 95% CI 1.08, 2.12,p = 0.016) but not associated with encouraging the child to be active, doing physical activity with the child, being active in front of the child or showing enthusiasm about being active.

**Conclusions:**

Most primary caregivers in the UK do not know how much physical activity is recommended for children but those who do may be more supportive of physical activity for their child. Wider dissemination of the guidelines could be an important step in increasing population levels of physical activity.

## Background

There is a wealth of evidence for the health benefits of regular physical activity participation in children [1]. However, less than a third of children in the UK are adequately active [2]. Low levels of physical activity in children are a particular public health concern because they may track into adulthood, potentially posing a risk to health throughout the life course [3].

The Chief Medical Office in the UK issues evidence-based physical activity guidelines which, along with information on sedentary behaviour and muscle-strengthening activity, include recommendations for the duration and intensity of physical activity needed for good health [4]. Prior to 2011, children were recommended to achieve at least 60 minutes of moderate-to-vigorous physical activity (MVPA) every day. However in 2011, age-specific guidelines superseded existing guidance, drawing on the latest evidence to recommend that children younger than 5 years who are capable of walking achieve a minimum of 180 minutes of any intensity activity a day and those aged 5 to 18 years achieve 60 minutes of MVPA a day. Achieving physical activity guidelines can help children to improve their cardiovascular health, support their development of co-ordination and movement skills, improve their bone health and maintain a healthy weight [4].

Theoretical models of behaviour change would suggest that knowledge of physical activity recommendations may influence behaviour [5–7]. In a sample of adults in Canada, physical activity was associated with knowledge of recommendations published as part of a national active living campaign [8], and active individuals in the USA were twice as likely to correctly identify government guidelines for physical activity as those who were inactive [9]. Similarly, data from the Health Survey for England in 2007 found that adolescent girls who knew the recommended level of physical activity for good health were substantially more likely to meet the recommendations than those without this knowledge; although this was not found for adolescent boys [10].

Primary caregivers can be closely involved in the activity levels of their children by providing support for active behaviours [11–13]. However, there is a trend for parents to overestimate the amount of physical activity their child achieves, incorrectly believing their child to be active [14]. Knowledge of physical activity recommendations for children could help parents to set accurate time-framed goals to achieve desirable physical activity behaviour in their child (e.g. specific, measureable, action-oriented, realistic and time-framed [SMART] goals [15]), supporting and modelling a physically active lifestyle [11, 14, 16].

Research carried out in the UK in 2007, 2011 and 2013 [17–19] found that while a third of adults believed they knew government physical activity guidelines for adults, only 18% were able to accurately recall even basic information contained within the guidelines. In a large sample of adolescents (11 to 15 years) surveyed in 2007, fewer than one in ten knew the physical activity guidelines for children and young people [17]. However, as far as we are aware, primary caregiver knowledge of child physical activity recommendations has only been examined once before, as part of a small feasibility study of Australian pre-schoolers [16]. This study found that only 20% of parents were aware of recommendations. The current study therefore assessed knowledge of paediatric physical activity recommendations among primary caregivers in a large, community-based, UK sample and examined associations between knowledge and parental support and modelling of physical activity.

## Methods

### Participants

Participants were parents and caregivers taking part in the Gemini twin birth cohort: a population-based sample of twins born in England and Wales in 2007. A total of 2,402 families participated in baseline data collection when the twins were approximately 8 months (36% of all families with twins born between March and December 2007). The Gemini sample is described in detail elsewhere [20]. Primary caregivers provided informed written consent and were free to withdraw from the study at any time without a given reason. Ethical approval for the Gemini study was granted by the University College London Committee for the Ethics of non-National Health Service Human Research.

1,113 primary caregivers (46% of baseline sample) took part in a home environment interview at wave 6 of data collection, when children were approximately 3.5 years old (40 months). Compared with the baseline Gemini sample, interview participants were slightly older (34 vs. 33 years at twins’ birth; p < 0.001), more were educated to university level (48% vs. 42%; p < 0.001), and fewer were from ethnic minority groups (5% vs. 7%; p = 0.04).

### Measures

A telephone interview assessing aspects of the home environment was conducted with the primary caregiver to collect data relating to physical activity and the home physical activity, food and media environment.

During the interview, knowledge of the physical activity recommendations was assessed with the question: “Do you know how many minutes of physical activity per day health professionals recommend for young children”. Responses were ‘yes/no’. Where participants said “yes”, they were prompted by an open-ended question to state the recommended minimum number of minutes. Responses were categorised as ‘correct’ if they were 60 minutes a day, as featured in recent national campaigns Change for Life [21] and At Least Five [22]. While this study was being carried out, new guidelines for children aged <5 years were issued [4]. As far as we are aware, these guidelines had not yet been widely disseminated even among health professionals. However, participants who responded that children needed 180 minutes of activity a day, as stated in the new guidelines, were also categorised as ‘correct’. All other answers (including overestimations and underestimations) were categorised as ‘incorrect’, although in practice estimations were rare; participants generally reported that they didn’t know. Participants who initially said they did not know recommendations were categorised as ‘incorrect’ and were not distinguished from those who incorrectly said they knew recommendations. If participants didn’t know, or were incorrect, then researchers informed them of the correct guidelines. Data on the validity and reliability of this question were not collected in the current study (test-retest reliability of the rest of the home environment interview was assessed but this was not possible with this question as ‘incorrect’ answers were corrected by the researcher). However, similar methods of measurement have been used successfully elsewhere [17, 18].

Maternal age, height and weight were self-reported by questionnaire at baseline. Maternal BMI was calculated using the formula: weight (kilograms)/height^2^ (meters). Maternal education and ethnicity and presence of a co-habiting partner (‘yes/no’) were recorded during the telephone interview. Education was categorised as: ‘none or basic school education’, ‘vocational or high school education’ and ‘university education’. Ethnicity was categorised as ‘white’ and ‘ethnic minority group’ due to the low representation of participants from minority groups. All socio-demographic variables other than presence of a co-habiting partner were measured with respect to the mother of the twins, even if the interview was completed by a partner or other caregiver.

Parental support of physical activity and parental modelling of physical activity (i.e. the demonstration of positive physical activity behaviour) were recorded during the telephone interview. Four items measured support (e.g. “How often do you or your partner tell your child that being physically active is good for his/her health”, measuring communication of positive physical activity messages to one’s child). Three items assessed parental modelling (e.g. “How often do you or your partner show your child how much you enjoy being active”). All parental support and modelling items were scored on a Likert scale (1 = never; 5 = very often). Continuous scores for overall levels of support and overall levels of modelling were calculated using a composite score from all items included in the respective scales. Binary scores for individual components: ‘low’ (1 = never; 2 = rarely; 3 = sometimes) and ‘high’ (4 = often; 5 = very often), were also calculated. The parental support scale has been shown to have good internal consistency (Cronbach’s alpha = 0.78) and excellent test-retest reliability (r = 0.81) [13]. The parental modelling scale has been used previously for the assessment of the Change for Life campaign in the UK, when it was shown to have good internal consistency (Cronbach’s alpha = 0.80; Croker et al., unpublished). Indeed, in a sample of 44 primary caregivers from this study, test-retest reliability was satisfactory for the parental support scale (ICC = 0.68; 95% CI = 0.48-0.81) and good for the parental modelling scale (ICC = 0.78; 95% CI = 0.62-0.87).

### Analyses

Descriptive statistics were used to describe the sample and to report the frequency of knowledge of physical activity recommendations. Chi-square and t-test analyses were performed to assess socio-demographic differences between participants who did and did not have knowledge of guidelines. Univariate and multivariate binary logistic regression analyses were used to examine the relationship between socio-demographic characteristics and knowledge. Independent t-tests investigated the relationship between knowledge of recommendations (‘yes/no’) and overall parental support and overall parental modelling of physical activity. Binary logistic regression analyses were conducted with knowledge of physical activity recommendations as the exposure and individual components of parental support of physical activity and parental modelling of physical activity as outcomes. All models were adjusted for socio-demographic characteristics. All analyses were carried out in SPSS v 20 using a significance level of p < 0.05.

## Results

Participant characteristics (n = 1,113) are shown in Table 1. The majority of participants were the mothers of the twins (96.0%), with a small number of fathers (3.3%) and one female caregiver (0.1%).

**Table 1:** **Participant characteristics by knowledge of recommendations (n** = **1,113)**

	Total sample N (% of total)	Knowledge N (% of category)	No knowledge N (% of category)	p-value
Maternal education				<0.001*
None/basis school	173 (15.5)	18 (10.4)	155 (89.6)	
Vocational/high school	403 (36.2)	83 (20.6)	320 (79.4)	
University	537 (48.2)	136 (25.3)	401 (74.7)	
Maternal ethnicity				0.100
White	1,055 (94.8)	229 (21.7)	826 (78.3)	
Ethnic minority group	58 (5.2)	8 (13.8)	50 (86.2)	
Co-habiting partner				0.356
No	76 (6.8)	13 (17.1)	63 (82.9)	
Yes	1,037 (93.2)	224 (21.6)	813 (78.4)	
Maternal age (years; mean [SD])	34.5 (4.77)	34.8 (4.49)	34.5 (4.84)	0.304
Maternal BMI (kg/m^2^; mean [SD])	24.8 (4.58)	24.7 (4.7)	24.9 (4.56)	0.556

^*^Significant at a p < 0.05 level.

Only 21.3% (n = 237) of participants knew how much physical activity was recommended for children. Of those who were educated to university level, 136 (25.3%) knew physical activity recommendations, compared with 83 (20.6%) of those educated to high school/vocational level and 18 (10.4%) of those educated to basic school level or less. Knowledge of physical activity recommendations was higher in white participants (n = 229; 21.7%) than in participants in ethnic minority groups (n = 8; 13.8%) and higher in co-habiting participants (n = 224; 21.6%) than in single participants (n = 13; 17.1%). The only significant difference between those who did and did not know recommendations was in maternal education (Table 1).

As shown in Table 2, participants with a university or high school/vocational education were significantly more likely to know recommendations than those with basic or no school education (university: OR = 2.82; 95% CI = 1.66-4.79, p < 0.001; high school/vocational: OR = 2.23; 95% CI = 1.29-3.85, p = 0.003). There were no differences in knowledge by maternal ethnicity, maternal age, maternal BMI and presence of a co-habiting partner. The multivariate model provided a good fit for the data and explained 2.9% of the variance in knowledge of recommendations for children (Nagelkerke R^2^ = 0.029).

**Table 2:** **Regression analyses with knowledge as the outcome (n** = **1,113)**

	Univariate			Multivariate ^a^		
	OR	95% CI	p-value	OR	95% CI	p-value
Maternal education						
None/basic school	1.00			1.00		
Vocational/high school	2.23	1.30 to 3.85	0.004*	2.23	1.29 to 3.85	0.003*
University	2.92	1.73 to 4.94	<0.001*	2.82	1.66 to 4.79	<0.001*
Maternal ethnicity						
White	1.00			1.00		
Ethnic minority group	0.58	0.27 to 1.24	0.157	0.64	0.30 to 1.38	0.255
Co-habiting partner						
No	1.00			1.00		
Yes	1.34	0.72 to 2.47	0.357	1.12	0.60 to 2.10	0.720
Maternal age (years)	1.02	0.99 to 1.05	0.303	1.02	0.98 to 1.05	0.328
Maternal BMI (kg/m^2^)	0.99	0.96 to 1.02	0.566	1.00	0.97 to 1.03	0.933

^*^Significant at a p < 0.05 level. ^a^Multivariate model for knowledge of child guidelines: Hosmer-Leveshow test: X^2^(8) = 3.191, p = 0.922. Classified 78.6% cases; explained 2.9% variance. Model: X^2^(6) = 20.937, p = 0.002.

### Associations between knowledge and parental support and modelling of physical activity

Knowledge of child recommendations was associated with higher odds of communicating positive messages about physical activity to your child (OR = 1.52; 95% CI = 1.12-2.06, p = 0.008), watching the child participate in physical activity (OR = 1.69; 95% CI = 1.11-2.55, p = 0.013), and showing the child you enjoy physical activity (OR = 1.51; 95% CI = 1.08-2.12, p = 0.016) (Table 3). Knowledge of recommendations was not related to other components of parental support or modelling of physical activity. Knowledge was not related to overall parental support of physical activity (p = 0.506) or overall parental modelling of physical activity (p = 0.157).

**Table 3:** **Regression analyses with knowledge as the exposure and parental support and modelling as outcomes (n** = **1,113)**

Knowledge	OR	Univariate 95% CI	p-value	OR	Multivariate ^a^ 95% CI	p-value
*Communicate positive messages about physical activity (support)* ^*b*^						
No	1.00			1.00		
Yes	1.55	1.15 to 2.08	0.004*	1.52	1.12 to 2.06	0.008*
*Encourage child to be active (support)* ^*b*^						
No	1.00			1.00		
Yes	0.92	0.61 to 1.38	0.673	0.94	0.62 to 1.42	0.754
*Do physical activity with child (support)* ^*b*^						
No	1.00			1.00		
Yes	0.93	0.69 to 1.27	0.663	0.97	0.70 to 1.33	0.835
*Watch child participate in physical activity (support)* ^*b*^						
No	1.00			1.00		
Yes	1.59	1.06 to 2.38	0.024*	1.69	1.11 to 2.55	0.013*
*Show child enjoy physical activity (modelling)* ^*b*^						
No	1.00			1.00		
Yes	1.53	1.10 to 2.13	0.011*	1.51	1.08 to 2.12	0.016*
*Active in front of child (modelling)* ^*b*^						
No	1.00			1.00		
Yes	1.20	0.89 to 1.62	0.230	1.18	0.87 to 1.61	0.286
*Show enthusiasm about being active (modelling)* ^*b*^						
No	1.00			1.00		
Yes	0.96	0.66 to 1.39	0.808	0.87	0.60 to 1.28	0.490

*Significant at a p < 0.05 level. ^a^Adjusting for maternal education, maternal ethnicity, maternal age, maternal BMI, co-habiting partner and other children in the home. ^b^No knowledge (reference category) versus knowledge for participants who often/very often provided component of parental support or modelling.

## Discussion

Only a fifth of primary caregivers in our sample knew the recommended amount of physical activity for children. Higher maternal education increased the likelihood of knowledge in primary caregivers and knowledge was related to components of parental support and modelling of physical activity: communicating positive messages about physical activity to the child, watching the child participate in physical activity and showing the child they enjoyed physical activity themselves.

Our findings are consistent with a small pilot from Australia which found that 20% of 44 parents knew the Australian physical activity recommendations [16]. As the recommended amount of physical activity is one of the key messages in UK government guidelines [23], our findings suggest that the information contained in government physical activity guidelines may not be reaching large sections of the population. Teamed with the possibility that most parents incorrectly believe their child to be active [14], this could be hampering efforts to increase levels of childhood physical activity.

Maternal education was closely related to knowledge of physical activity recommendations for children. A similar relationship has been reported elsewhere for knowledge of recommendations for adults [9, 19, 24, 25], and is consistent with evidence that education is an important correlate of physical activity [26]. Low levels of education may present a barrier to knowledge of recommendations which might manifest as poorer access to the information, differential salience of the message, or increased difficulty in retaining the information presented in the guidelines [27]. Because educational attainment is to a large extent non-modifiable, research is needed to identify best-practice methods of guideline design and delivery to disseminate information equitably. Simplifying the health message may aid effective dissemination. Recent research found that nine in ten adults in Scotland knew dietary recommendations in UK government guidelines which state that individuals should eat at least five portions of fruit and vegetables a day [18]. The contrast with our finding that only one in five primary caregivers knew physical activity recommendations for children might be in part due to the simplicity of the ‘5-a-day’ message. This message is easy to understand and remember and can therefore be used within the private sector to advertise the health benefits of products or be discussed by the media, increasing exposure to the message. Creating a simple message to accompany more detailed physical activity recommendations may therefore be beneficial.

Knowledge of recommendations was related to higher levels of some components of parental support and modelling of physical activity (likelihood of communicating positive messages about physical activity, watching your child participate in physical activity, and showing your child you enjoy physical activity), but not others (encouraging your child to be active, doing physical activity with your child, being active in front of your child and showing enthusiasm about being active to your child). These inconsistent findings indicate that further research is needed to ascertain whether knowledge alone has a functional role in increasing parental support and modelling of physical activity.

Additionally, the cross-sectional nature of this study cannot distinguish whether a relationship between knowledge and parental support and modelling is being driven by i) caregivers who are already motivated by physical activity seeking out relevant physical activity recommendations or ii) exposure to recommendations motivating caregivers to support and model physical activity for their children. However, the fact that knowledge was generally related to behaviours that promote physical activity without requiring the caregiver to actually be active themselves might support the latter explanation. Even if only part of the relationship is explained by exposure to recommendations leading to parental support and modelling, this is a potentially modifiable pathway to increase childhood activity levels [13–15].

There are some limitations to this study. Firstly, research is needed to improve the quality of measures of parental support and modelling. Secondly, as we did not have measures of actual child physical activity behaviour, we cannot gain insight into the relationship between primary caregiver knowledge of recommendations and physical activity in children. More detailed measurement of knowledge of physical activity recommendations would also have enriched the interpretation of findings. It may be advantageous to use future research to develop an understanding of whether caregivers who do not know physical activity recommendations tend to underestimate or overestimate recommended amounts of physical activity and the proportion of caregivers who inaccurately believe they know recommendations; this information was not recorded in the current study.

The over-representation of white participants and mothers is another limitation of the study. A relationship between ethnicity and knowledge of physical activity recommendations has been reported in some studies [9, 25] but not in others [19, 24]; any relationship may have been obscured in our results. Low representation of ethnic minority groups (5.2%) and fathers (3.3%) limits the generalisability of our results to other populations.

## Conclusion

This is the first study to estimate primary caregiver knowledge of physical activity recommendations included in government guidelines for children in the UK. Only one fifth of primary caregivers knew the recommended amount of physical activity for children and this is likely to be an overestimate as a result of a relatively highly-educated sample. Much more needs to be done to develop strategies to satisfactorily disseminate physical activity guidance, particularly among groups with less education. This could help caregivers foster beneficial physical activity levels in their children during a formative period of their lives.

## Abbreviations

CMO: chief medical office
SD: standard deviation
BMI: body mass index
ICC: intra-class correlation
OR: odds ratio
CI: confidence interval.
